# Cardiopulmonary resuscitation missed by bystanders: Collateral damage of coronavirus disease 2019

**DOI:** 10.1111/aas.14117

**Published:** 2022-08-05

**Authors:** Giuseppe Stirparo, Nazzareno Fagoni, Lorenzo Bellini, Aurea Oradini‐Alacreu, Maurizio Migliari, Guido Francesco Villa, Marco Botteri, Carlo Signorelli, Giuseppe Maria Sechi, Alberto Zoli

**Affiliations:** ^1^ Faculty of Medicine School of Public Health—University of Vita‐Salute San Raffaele Milano Italy; ^2^ Department of Research and Development Agenzia Regionale Emergenza Urgenza Headquarters (AREU HQ) Milano Italy; ^3^ AAT Brescia, Azienda Regionale Emergenza Urgenza (AREU), Department of Anaesthesia, Intensive Care and Emergency, ASST Spedali Civili University Hospital Brescia Italy; ^4^ Department of Molecular and Translational Medicine University of Brescia Brescia Italy

**Keywords:** cardiac arrest, COVID‐19, defibrillators, resuscitation, return of spontaneous circulation

## Abstract

**Objective:**

The coronavirus disease 2019 (COVID‐19) pandemic changed the time‐dependent cardiac arrest network. This study aims to understand whether the rescue standards of cardiopulmonary resuscitation (CPR) and out‐of‐hospital cardiac arrest (OHCA) were handled differently during pandemic compared to the previous year.

**Methods:**

Data for the years 2019 and 2020 were provided by the records of the Lombardy office of the Regional Agency for Emergency and Urgency. We analysed where the cardiac arrest occurred, when CPR started and whether the bystanders used public access to defibrillation (PAD).

**Results:**

During 2020, there was a reduction in CPRs performed by bystanders (odds ratio [OR] = 0.936 [95% confidence interval (CI_95%_) 0.882–0.993], *p =* .029) and in the return of spontaneous circulation (ROSC) (OR = 0.621 [CI_95%_ 0.563–0.685], *p <* .0001), while there was no significant reduction in the use of PAD. Analysing only March, the period of the first wave in Lombardy, the comparison shows a reduction in bystanders CPRs (OR = 0.727 [CI_95%_ 0.602–0.877], *p =* .0008), use of PAD (OR = 0.441 [CI_95%_ 0.272–0.716], *p =* .0009) and in ROSC (OR = 0.179 [CI_95%_ 0.124–0.257], *p <* .0001). These phenomena could be influenced by the different settings in which the OHCAs occurred; in fact, those that occurred in public places with a mandatory PAD were strongly reduced (OR = 0.49 [CI_95%_, 0.44–0.55], *p <* .0001).

**Conclusions:**

COVID‐19 had a profound impact on the time‐dependant OHCA network. During the first pandemic wave, CPR and PAD used by bystanders decreased. The different contexts in which OHCAs occurred may partially explain these differences.

**Editorial Comment**

This retrospective analysis of out‐of‐hospital cardiac arrest cases and bystander‐initiated life support in one region in Italy showed major differences during the first year of the COVID‐19 pandemic compared to a matched pre‐pandemic period.

## 1 INTRODUCTION

The coronavirus disease 2019 (COVID‐19) pandemic has had an impact on the epidemiology of out‐of‐hospital cardiac arrest (OHCA),^1^ indeed, the incidence^1^, ^2^, ^3^ and mortality^4^, ^5^, ^6^ of OHCAs increased in 2020. However, there is no clear evidence whether these phenomena are due to the major complications of COVID‐19 or to delayed response times and late arrival of ambulances in an attempt to save patients^7^, ^8^ or even to the lack of CPR training due to the logistical complications of the pandemic.^9^

Recent studies have shown that OHCAs increased during the pandemic period by up to 60% compared with 2019.^2^, ^10^ Furthermore, the increase in the number of OHCAs was followed by a decrease in the return of spontaneous circulation (ROSC) by up to 41%.^11^, ^12^ These trends are probably the consequences of a 10% lower number of CPRs performed by bystanders^13^, ^14^ prior to the arrival of ambulance and/or Advanced Life Support (ALS) vehicle, and the delay in emergency medical service (EMS) response due to reorganisation and profound stress.^15^, ^16^ In addition, the Italian Ministry of Health recommended that people should not enter Emergency Departments (EDs), but call the 112 emergency number, and patients often refused to be transported by ambulance to the hospital ED for fear of being infected.^17^, ^18^ Thus, the reluctance of patients to be transported to the ED during the pandemic period changed the epidemiology of time‐related diseases,^19^, ^20^ increasing the number of cardiac arrests occurring at home.^21^

Since the beginning of the pandemic, bystanders' fear of being infected while performing CPR has been a debated topic in the scientific community, especially among those involved in lay education.^22^, ^23^ Therefore, as chest compressions can produce aerosols, the chain of survival had to be modified to ensure a greater safety for bystanders. However, there has been a reduction in the number of CPRs performed by bystanders and in the use of public access to defibrillation (PAD).^24^, ^25^ Consequences include increased mortality after OHCA and a worst neurologic outcome for hospitalised patients.^26^

Lombardy was the region most affected by the COVID‐19 outbreak in Italy. During the first wave, through April 30, Lombardy had more than 75,000 verified positive cases, and 13,772 deaths out of the 27,967 (49.2%) that occurred throughout Italy.^8^ The aim of this article is to analyse the impact of the COVID‐19 pandemic in the Lombardy region, in particular: (i) the comparison between the number of CPRs performed by bystanders after OHCA in 2019 and 2020; (ii) the number of ROSC and the number of PAD used and (iii) the relationship between the ROSC and the CPR performed by bystanders.

## 2 METHODS

This is a retrospective observational cohort study. The study was conducted according to the principles of the declaration of Helsinki and was approved by the Regional Agency for Emergency and Urgency (Agenzia Regionale Emergenza Urgenza, AREU) Data Protection Officer (DPO) in June 2021. AREU's DPO supports the owner, employees and controllers in data retention and risk management according to the principles and indications of the European Rules. The AREU DPO is therefore the technical and legal advisor, with executive power, and the data was provided by the records of the Lombardy office of AREU after DPO approval.

### 2.1 Data registry

We analysed all records saved as “cardiac arrest” in Emergency Management (EmMa) database in 2019 and 2020. The label “cardiac arrest” is given by the healthcare personnel through 112 (Unique Emergency Number – NUE) based on the description of the bystander or the ambulance crew (the latter being rescuers qualified in Basic Life Support manoeuvres). The ambulance crew is trained to recognise cardiac arrest, which is defined as the absence of consciousness to a verbal and tactile stimulus, and the absence of breathing. This is enough to start Basic Life Support manoeuvres by rescuers. While bystanders begin external chest compression under the guidance of healthcare personnel through 112. Possible causes of cardiac arrest are evaluated by the medical team if an advanced vehicle with a doctor on board is sent to the event.

The data analysis process was conducted employing the Statistical Analysis System of AREU (SAS‐AREU portal). The portal contains all data related to emergency calls. All types of cardiac arrest, both medical and traumatic, were selected.

### 2.2 The Regional Agency for Emergency and Urgency

In the Lombardy region, AREU is responsible for pre‐hospital EMS. This region was the first in Europe to face with COVID‐19,^27^, ^28^, ^29^, ^30^ and consequently had to modify its emergency system.^30^ AREU coordinates and ensures territorial first aid by means of 265 ambulances with a crew of 2–3 rescuers qualified in Basic Life Support manoeuvres, 50 Intermediate Rescue Vehicles (ambulance or car) with a nurse, 59 Advanced Rescue Vehicles with a doctor qualified in ALS and 5 helicopters with ALS crew. All interventions carried out by EMS teams are recorded on the EmMa portal.^31^ Detailed description of the system can be obtained from literature.^8^, ^16^, ^32^

### 2.3 Statistical analysis

Continuous variables are presented as mean and SD while categorical variables are presented as numbers and percentages. The total number of OHCAs was analysed by means of *t*‐test for paired data.

The total number of CPRs performed by bystanders, the number of time bystanders used PAD and the number of ROSC were analysed by means of odds ratios (OR) against the total number of OHCAs; 95% confidence intervals (CI_95%_) were provided. Differences were considered significant when *p* < .05, otherwise they were considered nonsignificant. The statistical software Prism 8.0.1 (GraphPad Software LLC, San Diego, CA, USA) was used for this purpose.

## 3 RESULTS

Table 1 shows the total number of OHCAs that occurred and were recorded by the SAS‐AREU portal, and includes all types of cardiac arrest (traumatic and medical), the total number of CPRs performed by bystanders, the number of times bystanders used PAD, and the number of ROSCs per month. The analysis of the number of CPRs involves manoeuvres initiated by bystanders, prior to the arrival of the BLS ambulance team and/or ALS vehicle, and consist of external chest compressions with or without breathing manoeuvres. Manoeuvres also include the use of the PAD if present at the scene and if the bystanders is confident of automatic external defibrillation

**TABLE 1:** Number of OHCAs per month, CPRs performed by bystanders, number of PAD used and number of ROSC in pandemic (2020) and in the previous year

	Number of OHCAs		CPRs performed by bystanders		PAD used by bystanders		ROSC	
	2019	2020	2019	2020	2019	2020	2019	2020
January	1241	1113	268	225	28	25	84	68
February	1188	1058	272	213	23	28	82	61
March	1097	1767	247	308	40	29	126	40
April	940	1148	219	233	31	29	84	45
May	956	942	215	205	25	19	76	60
June	917	895	203	193	23	25	77	57
July	949	900	171	191	21	24	77	57
August	851	928	170	205	18	20	71	66
September	802	878	197	220	21	30	83	65
October	983	1223	229	298	22	40	82	75
November	1025	1290	247	283	36	42	96	74
December	1191	1230	267	254	40	29	98	64
Total	12,140	13,372	2705 (22.3%)	2828* (21.1%)	328 (2.7%)	340 (2.5%)	1036 (8.5%)	732* (5.5%)

Abbreviations: CPRs, cardiopulmonary resuscitations; OHCAs, out‐of‐hospital cardiac arrests; PAD, public access to defirbillation; ROSC, return of spontaneous circulation.

*p < .001 compared to 2019.

In 2020, there was a 9.2% increase in the total number of OHCAs compared to 2019 (*p* = .230), and in March 2020 there were more OHCAs than in 2019 (OR 1.53, CI_95%_ 1.41–3.32; *p* < .0001). We observed fewer OHCAs of traumatic origin in 2020 than 2019, 722 (5.40%) and 841 (6.93%), respectively (OR 0.676, CI_95%_ 0.692–0.850; *p* < .0001). Table 1 shows the monthly number of OHCAs. The total number of CPRs attempted by bystanders in 2020 is higher than in 2019, but the percentage of CPRs by bystanders on OHCAs decreased significantly in 2020 compared to 2019 (21.1% vs. 22.2%, *p* = .020). ROSC was lower in 2020 (8.5% vs. 5.5%; *p* < .0001) but the percentage of PADs used did not change (2.5% vs. 2.7%, *p* = .427). Although the total number of CPRs attempted by bystanders in 2020 is higher than in 2019, the percentage of CPRs by bystanders on OHCAs decreased significantly in 2020 compared to 2019 (21.1% vs. 22.2%, *p* = .029).

CPR, PAD, and ROSC were analysed against the total number of OHCAs; the OR and their 95% confidence intervals with relative significance are shown in Table 2. The data show a significantly lower probability of ROSC and receiving CPR from a bystanders in 2020 compared to 2019, and an even lower probability when considering March. The number of times bystanders used PAD was significantly lower in March 2020 compared to March 2019.

**TABLE 2:** Odds ratios of the events analysed [95% confidence intervals], and significance

	CPRs performed by bystanders	PAD used by bystanders	ROSC
2019 versus 2020	0.936 [0.882–0.993] *p =* .020	0.939 [0.806–1.096] *p =* .427	0.621 [0.563–0.685] *p <* .0001
March 2019 versus March 2020	0.727 [0.602–0.877] *p =* .0008	0.441 [0.272–0.716] *p =* 0.0009	0.179 [0.124–0.257] *p <* .0001
December 2019 versus December 2020	0.900 [0.742–1.09] *p =* .290	0.695 [0.428–1.128] *p =* .141	0.612 [0.442–0.848] *p =* .003

Abbreviations: CPRs, cardiopulmonary resuscitations; PAD, public access to defibrillation; ROSC, return of spontaneous circulation.

Table 3 shows the locations where EMS teams were sent to rescue people with OHCA. The OHCAs rescue at home increased significantly from 82.2% in 2019 to 87.3% in 2020 (OR 1.49, CI_95%_ 1.39–1.60; *p <* .0001).

**TABLE 3:** Place where cardiac arrests occurred in 2019 and 2020. “Others”: lake, sport facility, or data not available

Places where cardiac arrests occurred	2019	2020	Mandatory PAD
	*N* (%)	*N* (%)	
Home	9984 (82.2%)	11680 (87.3%)	No
Street	970 (8.0%)	797 (6.0%)	No
Extended care	422 (3.5%)	320 (2.4%)	Yes
Public offices	340 (2.8%)	80 (1.8%)	Yes
Work place	117 (1.0%)	83 (0.6%)	Yes
Mountain	53 (0.4%)	61 (0.5%)	No
Healthcare facilities	52 (0.4%)	45 (0.3%)	Yes
School	8 (0.06%)	5 (0.03%)	No
Others	194 (1.6%)	301 (2.6%)	
Number of OHCAs occurred in places with PAD	931 (7.7%)^a^	528 (3.9%)^a^	

Abbreviations: CPRs, cardiopulmonary resuscitations; CI_95%_, 95% confidence interval; OHCAs, out‐of‐hospital cardiac arrests; OR, odds ratio; PAD, public access to defirbillation.

^a^ OR 0.49, CI_95%_ 0.44–0.55; *p* < .0001.

**FIGURE 1:**
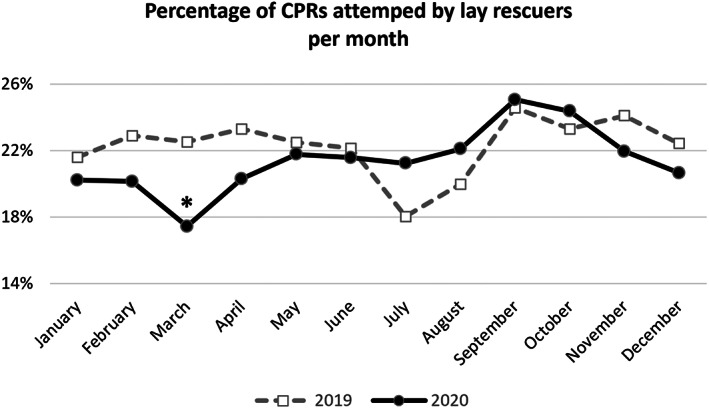
Percentage of cardiopulmonary resuscitations (CPRs) attempted by bystanders per month in 2019 (dashed grey line) and 2020 (continuous black line). *p < .001.

Figure 1 shows the percentage of CPRs attempts by bystanders per month. During the first outbreak in Italy (March 2020 vs. March 2019, 17.4% vs. 22.5%, respectively; *p <* .001), CPR was performed less than in 2019; this finding was similar during the second outbreak in Italy but not significant (December 2020 vs. December 2019, 20.6% vs. 22.4%, respectively; *p* = .290).

**FIGURE 2:**
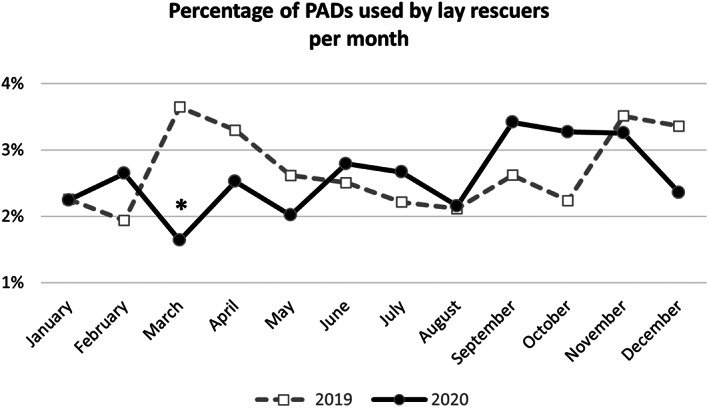
Percentage of public access to defibrillation (PAD) used by bystanders during cardiopulmonary resuscitation (CPR) in 2019 (dashed grey line) and 2020 (continuous black line). *p < .001.

Figure 2 shows the percentage of PAD used out of the total number of OHCAs. In 2020, the number of time bystanders used PAD was not different compared to 2019 (OR 0.939, CI_95%_ 0.806–1.096; *p* = .430). There was a significant reduction in PAD used by bystanders in March 2020 compared to March 2019 (OR 0.441, CI_95%_ 0.272–0.715; *p <* .001).

## 4 DISCUSSION

Among the studies on OHCAs in the COVID‐19 era, this is one of those analysing one of the largest groups of recorded cases, with more than 10,000 cardiac arrest per year. Despite a non‐significant 9.2% increase in the total number of OHCAs in 2020, the Lombardy region showed a higher number of OHCAs in March 2020 compared to 2019, probably as a result of a higher rate of cardiac arrests in COVID‐19 patients.^1^, ^2^, ^3^ The OHCAs increase is consistent with available literature, +61% in 2020 compared to 2019 and may be explained by the increased risk of OHCA in COVID‐19 patients or the reduction in the number of ST‐elevation myocardial infarction diagnoses related to the hesitation of spontaneous presentation in ED.

Furthermore, the rate of bystanders CPRs and the ROSC rate decreased significantly between 2019 and 2020, particularly during the first wave of the pandemic. In fact, there was a significant reduction in CPRs attempted by bystanders in 2020 compared to 2019, with the largest reduction occurring in March 2020 (17.4% vs. 22.5%), during the first outbreak. These data are consistent with Scquizzato et al.^4^ and Marijon et al.,^12^ who found a reduction in CPRs attempted by bystanders. This phenomenon could be related to a psychological factor. Bystanders might be less inclined to initiate CPR on a stranger with OHCA for fear of possible COVID‐19 infection, as pointed out by Kapoor et al.^23^

We also found a reduction in OHCA in public areas where the PADs were available in 2020 compared to 2019. This reduction is particularly significant, as in 2020 the number of OHCAs occurring in places where PAD was present was 3.9%, compared to 7.7% in 2019. The reason, most likely, is due to the fact that, especially during the pandemic waves, many workers were smart working and many companies were closed, as well as public offices, which in Italy are the places where PADs are most often present. Despite this situation, the number of PADs used did not differ between 2020 and 2019, although in March 2020 the use of PADs was significantly lower than in the same month in 2019, which underlines the fact that the closures of places where PADs are present actually affected their use. We cannot exclude that this phenomenon was due to media awareness related to cardiac arrest and its treatment that occurred in 2020 as a result of the pandemic compared with the previous year. If this was the case, the public was sensitized to pay more attention to the problem, and it is likely that when PAD could be used, this occurred more frequently than in the past. Despite this, ROSC decreased significantly in 2020, which is consistent with the literature.^1^

In March 2020, we registered the largest reduction in ROSC compared to 2019; this phenomenon can be explained by the reduction in attempted CPRs and PADs used by bystanders. We cannot exclude that the location of cardiac arrests may play a central role, as we found an increase in OHCA occurring at home in 2020 than in 2019 (87.3% vs. 82.2%, respectively), where people are usually alone and away from PAD. Indeed, Table 3 shows the locations where PAD equipment is mandatory. The probability of having a cardiac arrest in locations with PAD was lower in 2020 than in 2019 (OR 0.49, CI_95%_ 0.44–0.55; *p <* .0001).

The differences in CPRs and PAD was not found when considering the second wave in December. While the number of CPRs performed by bystanders and PADs use in March 2020 were found to be different from March 2019, in December the data were not different between the 2 years. One explanation for this phenomenon could be that there were more OHCAs in March 2020 than in March 2019, whereas in December the 2020 data were consistent with the previous year; in addition, after the first wave, the approach to COVID‐19 patients changed and the fear of contact between individuals gradually decreased.

A retrospective analysis of the clinical management of OHCA would be a useful tool to understand which variables in the chain of survival may influence the possibility of ROSC. This analysis goes beyond the single pandemic wave and aims to record epidemiological changes in the time‐dependent OHCA network. In the literature, there are models that can predict ROSC based on several variables. For example, the Utstein‐based ROSC score was derived using the following variables: age, aetiology, location, assisted OHCA, bystander CPR, time to EMS arrival, and attachment rate.^33^, ^34^, ^35^ The presence of all the variables described would have allowed us to use the model mentioned above and to do further investigation and analysis on the ROSC data. Unfortunately, the lack of some variables does not allow further investigation that might be done in future studies.

The study found several changes strongly associated with pandemic peaks, thus achieving the main aim of the study. However, a limitation of the study is the possible reduced accuracy in the data collecting process due to the high pressure and stress of the emergency phase. Examining the impact of COVID‐19 on time‐dependent networks becomes essential to fully understand the collateral damage of the pandemic.

## 5 CONCLUSIONS

The COVID‐19 epidemic profoundly changed the epidemiology of OHCA. The possibility of ROSC decreased on an annual basis and reached a minimum during the first pandemic wave. Bystanders‐initiated CPR decreased significantly, and the lowest point was registered in March 2020, while use of PAD by bystanders unchanged. COVID‐19 pandemic modified the time‐dependent OHCA network, and this phenomenon can be included among its collateral damage.

## AUTHOR CONTRIBUTIONS

**Giuseppe Stirparo**: conceptualization, methodology, data curation, resources, visualization, reviewing and editing, statistical analysis, writing and original draft preparation. **Nazzareno Fagoni**: conceptualization, methodology, data curation, resources, visualization, reviewing and editing, statistical analysis, writing and original draft preparation. **Lorenzo Bellini**: writing and original draft preparation, reviewing and editing. **Aurea Oradini‐Alacreu**: writing and original draft preparation, reviewing and editing. **Maurizio Migliari**: resources, reviewing and editing. **Guido Francesco Villa**: resources, reviewing and editing. **Marco Botteri**: Resources, reviewing and editing. **Carlo Signorelli**: resources, reviewing and editing. **Giuseppe Maria Sechi**: resources, reviewing and editing. **Alberto Zoli**: resources, reviewing and editing.

## FUNDING INFORMATION

There are no funders to report for this submission.

## CONFLICT OF INTEREST

The authors declare no conflict of interest.
